# Enhanced Plasmonic Resonance Characteristics of AgNRs–Gold Film Hybrid System

**DOI:** 10.3389/fchem.2020.553541

**Published:** 2021-01-21

**Authors:** Yanping Yin, Jin Zhu, Zaoji Wang, Guojun Ma, Huining Yuan, Xiaolong Li

**Affiliations:** College of Electronic and Information, Jiangsu University of Science and Technology, Zhenjiang, China

**Keywords:** silver nanorod monomer, gap distances, nanorods dimer, figure of merit (FOM), plasmonic nanoruler

## Abstract

In recent years, the plasma gap resonance maintained by metal-film-coupled nanostructures has attracted extensive attention. This mainly originates from its flexible control of the spectral response and significantly enhanced field strength at the nanoparticle–film junction. In the present study, the tunability of local surface plasmon resonances (LSPRs) of nanorods coupled to a gold film is studied theoretically. To this end, the plasmonic resonances in the nanostructure of individual silver nanorod–gold film (AgNR-film) with different parameters are investigated. Obtained results show that the refractive index sensitivity (S) of nanostructures to the environment increases as the aspect ratio (*A*_*r*_) of nanostructures increase. It is found that when the aspect ratio (*A*_*r*_) is set to 3.5, the figure of merit (FOM) is the highest. Moreover, the variation in the gap distances of the nanorod monomer–gold film, electric field distribution of nanorods dimer, and the corresponding impact on the gold film are studied. It is concluded that the gap size of nanostructures has an exponential correlation with the resonance wavelength. Considering the remarkable influence of the gap size and the surrounding medium environment on the spectral shift of AgNR-film nanostructures, potential applications of the structure as a refractive index sensor and biomolecule measurement are proposed.

## Introduction

When the external light field illuminates the structure of the individual nanoparticle–gold film, it induces the collective oscillation of free electrons on the surface of the structure. When the frequencies of the light and electron oscillation match, the resonance will be formed. In this case, the structure is called the distinct resonant, and the corresponding spatial characteristics can significantly enhance the intensity of the near-field interaction by limiting the light to a deeper subwavelength range. Generally, the optical responses of plasma nanostructures remarkably depend on the geometry, symmetry, and topology of the constitutive elements, which lead to many technologically relevant optical phenomena. These phenomena include the plasmon-enhanced non-linear refraction and absorption (Sipe and Boyd, 1992; Kohlgraf-Owens and Kik, 2009), a biomedical imaging contrast agent (Loo et al., 2005; Huang et al., 2006), plasmon-enhanced fluorescence (Ditlbacher et al., 2002; Bakker et al., 2008), and the surface-enhanced Raman spectroscopy (SERS) (Moskovits, 1985; Campion and Kambhampati, 1998). Studies show that nanoparticles with anisotropic geometry obtain shape-induced spectral characteristics (Khan et al., 2016). Unlike the single absorption peak of the rotationally symmetric nanosphere, the anisotropic nanorods exhibit two peaks (Chen et al., 2015); strong incidence anisotropy is shown in the spectral positions excited by longitudinal plasma modes (electron oscillations parallel to the length of the nanorod) and transverse plasma modes (electron oscillations perpendicular to the length of the nanorod) (Habteyes and Terefe, 2014). The metal nanoparticles placed at the subnanometer separations from a gold film exhibit different plasma behaviors compared with equivalent independent nanoparticles, which originates from the symmetry breaking of the dielectric environment caused by a gold film. More specifically, the free electrons in the metal nanoparticles interact with the image charges in the gold film, resulting in the hybridization of various plasma modes. This causes large plasma shifts and occurrences of new plasmon resonance.

The simplest forms of nanospheres and a metal film have been thoroughly investigated, demonstrating that such near-field interactions exhibit complicated resonance characteristics. In this regard, it is believed that the field between the anisotropic nanorods and a gold film should exhibit more complicated resonance structures. Silver is the plasmon material with the lowest loss in visible light region, and compared with other silver nanoparticles, silver nanorods have the highest response sensitivity to the surrounding environment (Liu and Huang, 2013). Moreover, scholars applied the discrete dipole approximation (DDA) method to investigate the optical properties of nanorods (Brioude et al., 2005; Lee and Elsayed, 2005). Obtained results showed that the Raman signal enhancement effect of silver nanorods is better than that of gold nanorods, and the corresponding spectrum is narrower than that of gold nanorods (Schmucker et al., 2010; Mahmoud and El-Sayed, 2013). By changing the size of nanorods and the surrounding medium environment, it can achieve the artificial regulation of the position of the longitudinal surface plasmon resonance peak from the visible region to the near-infrared region. The nanogap between the particle and the film is defined as hot spots with a very high field concentration, which indicates plasmon-mediated optical sensing and enhancement applications. The difference in material properties between silver and gold nanorods leads to the higher electromagnetic field intensity around the silver nanorods. Accordingly, the sensitivity of silver nanorods to the surrounding environment is higher than that of gold nanorods. The enhancement factor of silver nanorods used for the near-field enhancement is 3.5–6.8 times more than that of gold nanorods (Jiang et al., 2012). Reviewing the literature indicates that the majority of performed investigations so far have focused on the metal films composed of single nanoparticles. Meanwhile, in the monomer–film system, the interaction between the dipolar plasma mode maintained by nanoparticles and the underlying film and the tuning mechanism of the plasma mode controlled by the gap distance have been extensively studied. However, few investigations have been carried out so far about the influence of underlying metal films on the coupled plasma modes maintained by complex nanostructures (Chen et al., 2015). More specifically, there is a lack of understanding of the plasma characteristics, including resonance position and radiation behavior of the metal-film-coupled nanoparticle clusters such as dimers or more complex nanostructures. Accordingly, investigating the potential applications correlated to these interesting systems are highly demanded.

In the present study, it is intended to perform a finite element numerical simulation to investigate the radiation features of the plasmonic gap modes in the gold-film-coupled nanorod monomer and dimers with nanometric nanorod–film gap distances. To this end, the extinction properties of nanorod monomer–film structures are initially analyzed with different aspect ratio (${\;A}_{r}=\frac{L}{2R}$), nanorod length (*L*), and nanorod radius (*R*). Then, the environmental refractive index (*n*_*a*_) is changed, and the correlation between the refractive index sensitivity (*S* = Δλ/Δ*n*_*a*_) (Miller and Lazarides, 2005; Lee and El-Sayed, 2006; Khan et al., 2016) is studied. Moreover, the nanosystem with the highest figure of merit (FOM = S/FWHM) (Sherry et al., 2005) will be determined accordingly. Second, the polyelectrolyte (PE) is used as the spacer layer in the highest FOM system to investigate the rule between resonance wavelength and the thickness of the PE layer. Finally, the effect of dimers in two different arrangements on the extinction and radiation is observed by the varying electric field direction.

## Numerical Simulation

**Figure 1:**
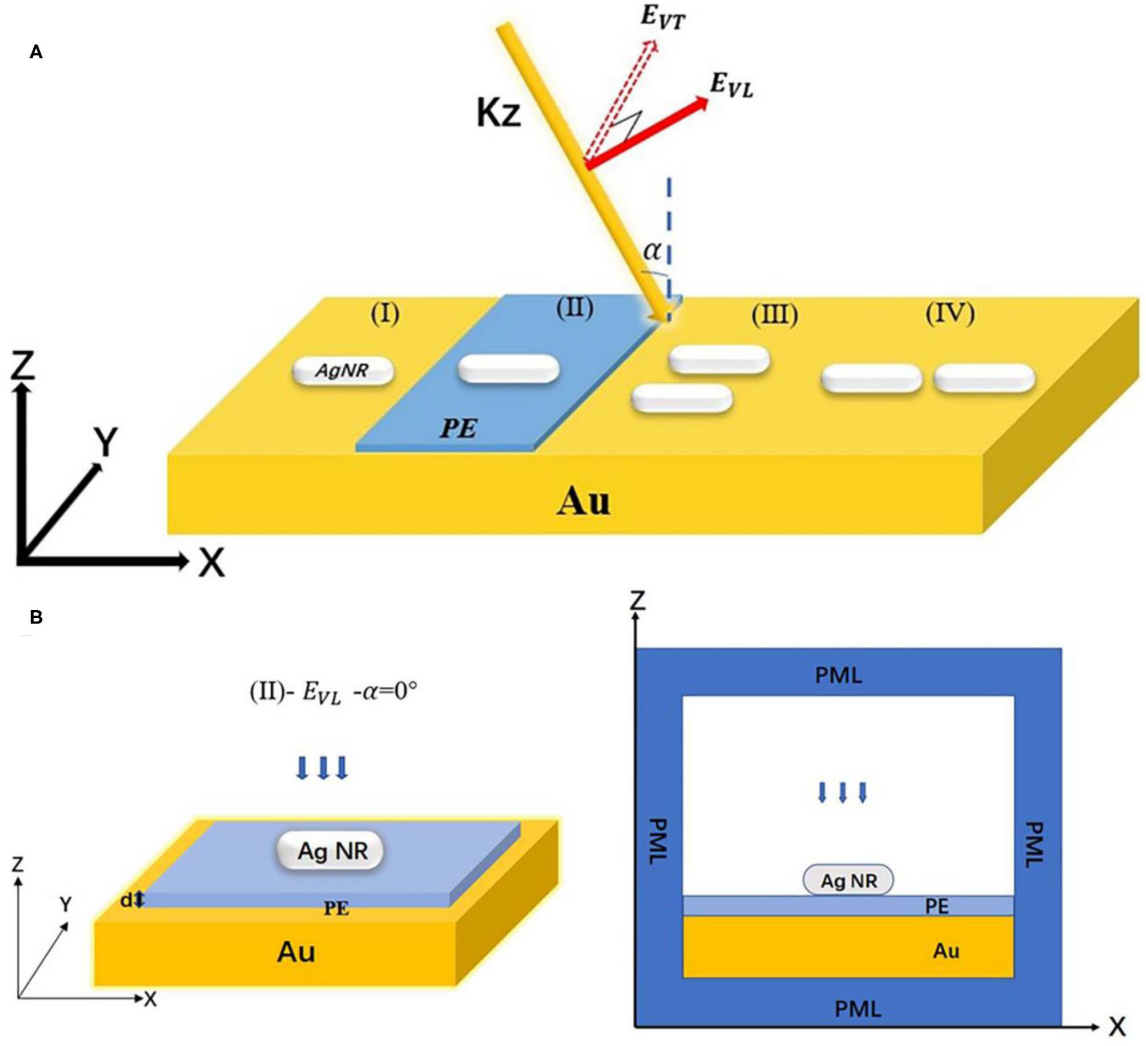
**(A)** Schematic configurations of three different incidences of light for illuminating the nanorod monomer and the nanorods dimers: ($E_{VL}-\alpha =0^{o};E_{VL}-\alpha =70^{o};E_{VT}-\alpha =0^{o}$) (I) silver nanorod monomer is suspending at 1.5 nm above the gold film. (II) Monomer–PE–gold film structure. (III) and (IV) Side-by-side and end-to-end dimer of nanorods. **(B)** is one of the simulation models in Panel **(A)**, (left) is the 3D model of (II) silver nanorods dimer–PE–gold film, and (right) is the x–z cross-section of numerical simulation.

In the present study, the spectrum and electric field distributions of AgNRs film structure are numerically simulated through COMSOL Multiphysics, which is a widely adopted commercial finite element software. The most basic idea of finite element method (FEM) was put forward by R.L. Courant in 1954 (Uranus and Hoekstra, 2004). Based on the variational principle and subdivision interpolation, boundary value problems are solved in FEM by discretizing the corresponding equations. The main idea in this method is to discretize the continuous solution space into discrete subdomains with relatively simple geometry. In each subdomain, the unknown field function of the whole solution domain is simulated by an approximate function, which can be obtained by interpolating the field function values of each node. In this way, an unknown quantity in the whole solution domain is converted to a field function value of each node. Accordingly, the initial continuous problem is converted to a problem of finite degrees of freedom. As long as these unknowns are solved, the approximate solution of the whole domain can be obtained by an interpolation method. It is worth noting that the accuracy and the total number of degrees of freedom in the FEM are determined by the number of subdomains. When the convergence conditions are satisfied, the approximate solution converges to the exact solution. In the simulation, the existence of gold film breaks the uniformity of the background field of the nanorod; the background field method is used to calculate the extinction cross-section and electric field distribution in two steps. In the first step, the Fresnel equation is employed to calculate the dielectric layers structure, including the air layer (*n*_*a*_ = 1), and the gold film (Johnson and Christy, 1972); in Figure 1A (II), there is also a spacer polyelectrolyte layer (PE, *n*_*b*_ = 1.5) (Mock et al., 2008). In the second step, silver nanorods (Johnson and Christy, 1972) are set to the active state, and the obtained background field in the first step is used as the excitation source to calculate the scattering and absorption of nanorods and set periodic conditions around the model. Nanorods are modeled as cylinders with a hemisphere at each end. In this regard, the aspect ratio is defined as ${\;A}_{r}=\frac{L}{2R}$. In all simulations, the nanorod radius is set to 15 nm, while the nanorod length (*L*) varies from 30 to 150 nm; when *L* = 30 nm, it is a nanosphere. Meanwhile, the length and width of the gold film are 400 nm with a thickness of 45 nm. AgNRs are coated with cetyltrimethyl ammonium bromide (CTAB) with a thickness of 1.5 nm. It should be indicated that CTAB is a type of surfactant. In order to simplify the model, the effect of surfactant is neglected in the present study. Since no gap material is considered in the simulation, it is assumed that silver nanorods are suspended at a height of 1.5 nm above the gold film. In order to improve the convergence rate of the numerical simulation, the calculation area is surrounded by perfectly matched layers (PMLs) with a thickness of 100 nm to reduce the reflection. Meanwhile, the simulation region is meshed with a minimum size of 0.5 nm. When the light beam incidents vertically (α = 0°), the plane wave can be divided into two kinds, including the electric fields along the long axis (*E*_*VL*_) and along the short axis (*E*_*VT*_) of nanorods. It should be indicated that the vertical–longitudinal (VL) mode and vertical–transverse (VT) mode of the plane wave do not contain the electric field component Ez. When the light beam is not perpendicular to the plate (α ≠ 0°), VL and VT modes of the plane wave contain the electric field component Ez. With these descroptions, α is the incident angle, and *E*_*VL*_ and *E*_*VT*_ denote azimuthal angles, which are set to 0° and 90°, respectively. Figure 1A presents a schematic diagram of the nanorod monomer, dimers film, and incident waves. Moreover, Figure 1B shows one of the simulation models in Figure 1A (II).

Taking Figure 1A (II) as an example to explain the interaction process between light and nanostructure, it can be divided into three consecutive steps (Wirth et al., 2014): (1) when the light wave irradiates on the dielectric layers (PE/gold film), thereby forming a specific electromagnetic field in the space layer; (2) the generated electromagnetic field interacts with nanorods and induces the light scattering; and (3) the scattered light from nanorods interfere with the reflected light from the space layer. In the gold film, the transmission light can be ignored because the thickness of the gold film is 45 nm, which is larger than the penetration depth of the light beam in the gold layer (about 25–30 nm) (Groeblacher et al., 2007).

When the light beam incidents are vertical (α = 0°), resonant modes *E*_*VL*_ and *E*_*VT*_ change with the aspect ratio of nanorods that can be calculated through the analytical expression of the Gans theory (Link et al., 1999). The extinction coefficient of particles corresponding to Gans theory is defined as follows:

(1) $$\begin{array}{l} \gamma =\frac{2\pi }{3\lambda }NV\varepsilon_{m}^{\frac{3}{2}}\underset{j}{\sum }\frac{\left(\frac{1}{P_{j}^{2}}\right)\varepsilon_{2}}{{\left(\varepsilon_{1}+\frac{1-P_{j}^{2}}{P_{j}}\varepsilon_{m}\right)}^{2}+\varepsilon_{2}^{2}} \end{array}$$

where *NV* is the product of the number of particles and the volume of a single particle. ε_*m*_ is the dielectric constant of the surrounding medium, and ε_1_ and ε_2_ are the real and imaginary parts of the dielectric constant of the particle itself, which is related to the wavelength λ of the incident light. Meanwhile, *P*_*j*_ is the factor related to the aspect ratio of nanorods along the three axes. Since rod-shaped particles have biaxial symmetry in shape, both resonance modes of Equation (1) are applicable to analyze the variation in the plasma resonance peak. Assuming the lengths of the three axes of nanorods as *L, D*, and *D*, where *D* = 2*R* and *L* > *D*, then the aspect ratio can be expressed in the form below:

(2) $$\begin{array}{l} P_{L}=\frac{1-e^{2}}{e^{2}}\left(-1+\frac{1}{2e}ln\frac{1+e}{1-e}\right) \end{array}$$

(3) $$\begin{array}{l} P_{D}=\frac{1-P_{L}}{2} \end{array}$$

where *e* = (1 − *D*^2^/*L*^2^)^1/2^, and $A_{r}=\frac{L}{2R}=\frac{L}{D}$, so $e={\left(1-\;1/{A_{r}}^{2}\right)}^{1/2}$.

In the numerical simulation, scattering, absorption, and attenuation cross-sections of the particle on the substrate are computed. The scattering cross-section is defined as the following:

(4) $$\begin{array}{l} \sigma_{sc}=\frac{1}{I_{0}}\int \int \left(n\cdot S_{sc}\right)dS \end{array}$$

where ***n*** is the normal vector pointing outwards from the nanorod. Moreover, *S*_*sc*_ and *I*_0_ denote scattered and incident intensities, respectively. The integral is taken over the closed surface of the scatterer. The absorption cross-section can be written in the form below:

(5) $$\begin{array}{l} \sigma_{abs}=\frac{1}{I_{0}}\int \int \int QdV \end{array}$$

where *Q* is the power loss density in the particle, and the integral is taken over its volume. Then, the extinction cross-section can be simply obtained from summation of these two terms:

(6) $$\begin{array}{l} \sigma_{ext}=\sigma_{abs}+\sigma_{sc} \end{array}$$

## Results and Discussion

**Figure 2:**
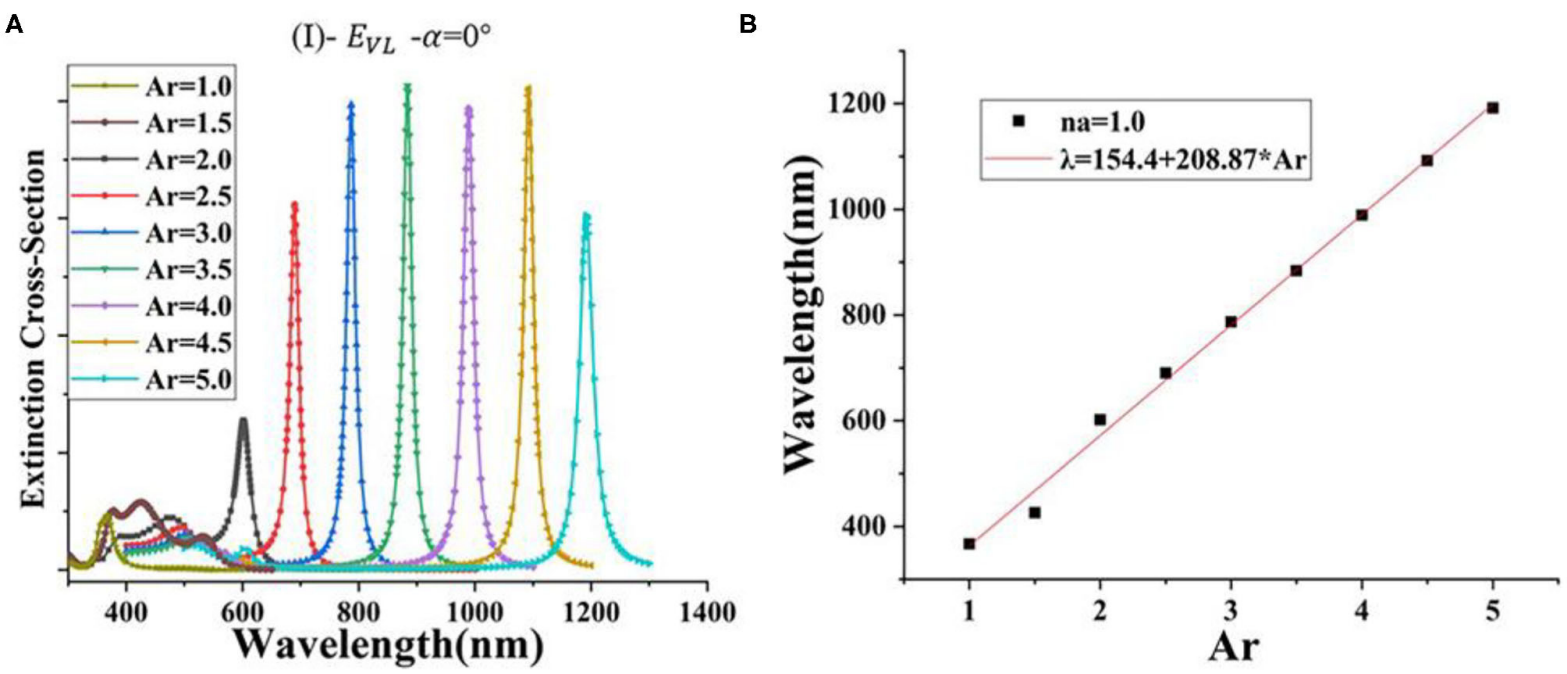
**(A)** The extinction cross-section of AgNR-film with different *A*_*r*_ in the air (*n*_*a*_ = 1); **(B)** correlation between *A*_*r*_ and the resonance peak position (λ) in the air (*n*_*a*_ = 1).

In this section, the extinction cross-section is normalized. First, the incidence angle is set to α = 0°, while the electric field is *E*_*VL*_, indicating an azimuth angle of 0°. Then, *A*_*r*_ of the silver nanorod is changed from 1 to 5 evenly. Figure 2A shows the obtained results in this regard. It is observed that the full width at half maximum (FWHM) of the extinction cross-section of the AgNR-film system with *A*_*r*_ = 2–5 is narrow and has strong and weak peaks at longitudinal and transverse resonance peaks, respectively. The long wave resonance peak shows strong anisotropic polarization characteristics related to the long axis resonance. It should be indicated that this trend is similar to that reported by other researchers (Johnson and Aikens, 2009; Guidez and Aikens, 2012). The resonance peak shifts from 600 to 1,200 nm. When *A*_*r*_ = 1.5, three waves appear in the extinction cross-section, while its intensity is lower than that of the system with *A*_*r*_ = 2–5. Moreover, we found that there is a peak at 367 nm for the nanosphere and a very small peak near the wavelength of 500 nm during the simulation. The extinction cross-section of the AgNR-film structure with an aspect ratio of 2–5 presents a weak and a strong peak, the nanostructure with an aspect ratio of 1.5 presents three peaks, and the nanosphere–gold film (AgNR-film) structure presents a strong and a weak peak. This can be be regarded as the transition from a AgNR-film structure to a AgNR-film structure.

Compared to the individual nanorod, in the AgNR-film structure, when the distance between the nanorod and film is very small, the longitudinal resonance of the nanorod produces a reverse parallel dipole (actually a quadrupole mode) on the gold film. Because the opposite direction energy will cancel each other (the built-in field of the nanorod will be weakened), the resonance frequency of the nanostructure will be reduced (wavelength redshift). It can also be seen from the simulation results in Supplementary Figure 1.

Figure 2B shows the wavelength of the highest peak corresponding to different *A*_*r*_ models in Figure 2A. A curve fitting process is performed to obtain a mathematical correlation between the aspect ratio *A*_*r*_ and the resonance wavelength λ. It is observed that the redshift linearly increases as *A*_*r*_ increases:

(7) $$\begin{array}{l} \lambda =154.4+208.87^{*}A_{r} \end{array}$$

**Figure 3:**
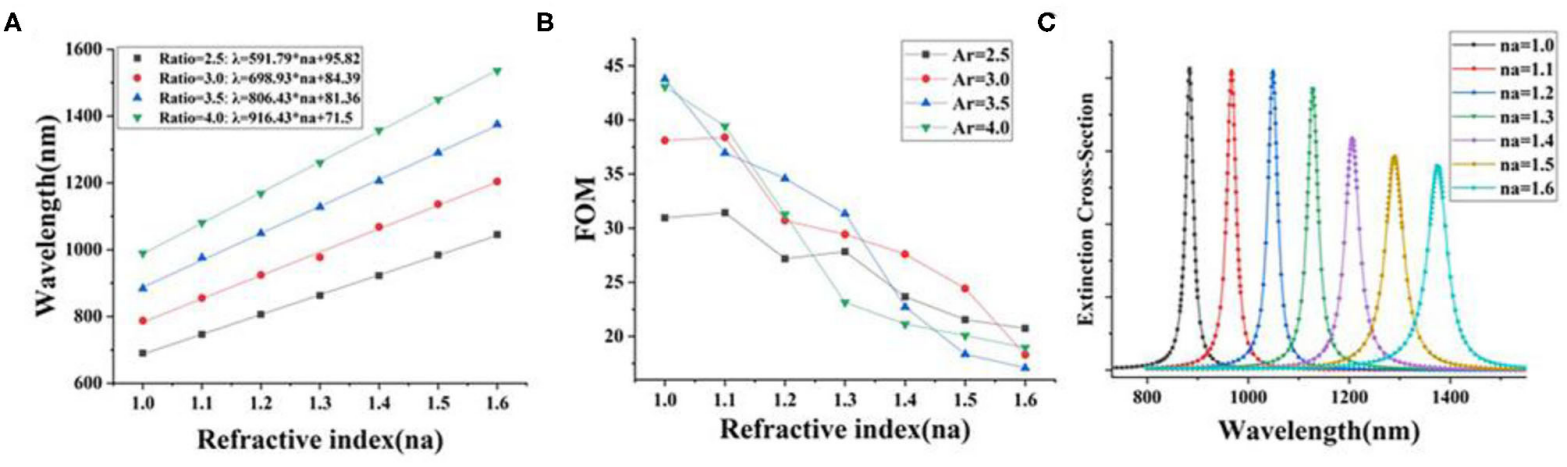
**(A)** The extinction cross-section peak position of AgNR-film structure with different *A*_*r*_ values and different surrounding refractive indices (*n*_*a*_); **(B)** the figure of merit (FOM) of AgNR-film structure with different *A*_*r*_ values in different surrounding refractive indices (*n*_*a*_); **(C)** extinction cross-section of the AgNR-film structure for *A*_*r*_ = 3.5 with different surrounding refractive indices (*n*_*a*_).

Figure 3 illustrates that *A*_*r*_ of the AgNR-film structure is set from 2.5 to 4 in different surrounding refractive index (*n*_*a*_) of 1–1.6. Figure 3A shows that the position of the extinction cross-section peak changes linearly as the surrounding environment refraction index (*n*_*a*_) changes under the same *A*_*r*_. Moreover, it is found that the resonance wavelength of nanostructures with different *A*_*r*_ grows at different rates as the environment changes. According to the definitions of slope (Δλ/Δ*n*_*a*_) and environmental sensitivity (S = Δλ/Δ*n*_*a*_), the refractive index sensitivity (S) is the slope of the linear fitting function of nanorods in Figure 3A. The corresponding refractive index sensitivity (S) for *A*_*r*_ = 2.5, 3.0, 3.5, and 4.0 are 591.79, 698.93, 806.43, and 916.43 nm/RIU, respectively, where RIU stands for the refractive index sensitivity unit. The larger the *A*_*r*_ of the silver nanorod, the greater the refractive index sensitivity (S) (Miller and Lazarides, 2005; Khan et al., 2016). In order to obtain the correlation function among aspect ratio (*A*_*r*_), environmental refractive index (*n*_*a*_), and resonance wavelength (λ), the conventional method (Link et al., 1999) can only express the correlation between the aspect ratio, dielectric constant of the uniform background field, and resonance wavelength. Therefore, the method of three-dimensional curve fitting is used in this paper for calculating the AgNR-film. The following expression is obtained:

(8) $$\begin{array}{l} \lambda =157.17-20.39*A_{r}+33.37*n_{a}+219.9*A_{r}*n_{a} \end{array}$$

When *n*_*a*_ = 1, the relationship between aspect ratio (*A*_*r*_) and resonance wavelength (λ) can be obtained by Equation (8): λ = 190.54 + $199.51^{*}A_{r}$. In Figure 2A, when *n*_*a*_ = 1, the relationship between aspect ratio (*A*_*r*_) and resonance wavelength (λ) is Equation (7): λ = 154.4 + $208.87^{*}A_{r}$. Based on Supplementary Table 2, it is found that when the aspect ratio *A*_*r*_ is set to 2.5–4, the deviation between Equations (7, 8) and the simulation results is not more than 2%.

In order to find the nanostructure with the highest figure of merit to carry out the following simulation, we introduced the parameter FOM. As the sensitivity (S) increases, the corresponding FOM does not necessarily increase because the FOM is related to the full width at half maximum (FWHM), but the variation of FWHM is irregular (see Supplementary Figure 3). It should be indicated that FOM is the ratio of refractive index sensitivity (S) to the FWHM of the LSPR peak (FOM = S/FWHM) (Sherry et al., 2005). Figure 3B illustrates the FOM of AgNR-film structure with different aspect ratio (*A*_*r*_) in different surrounding refractive indices (*n*_*a*_). It is found that for the AgNR-film with *A*_*r*_ = 3.5 in the air (*n*_*a*_ = 1), the system with the highest FOM can be achieved.

In recent years, nanorods have emerged in LSPR-based sensing and detection applications (Chen and Ming, 2012), which mainly originates from their easy preparation. The larger the aspect ratio (*A*_*r*_) in the AgNR-film structure, the more sensitive it is to the variation of the refractive index of the surrounding medium. Considering this superior characteristic of the LSPR, it can be used as a refractive index sensor.

**Figure 4:**
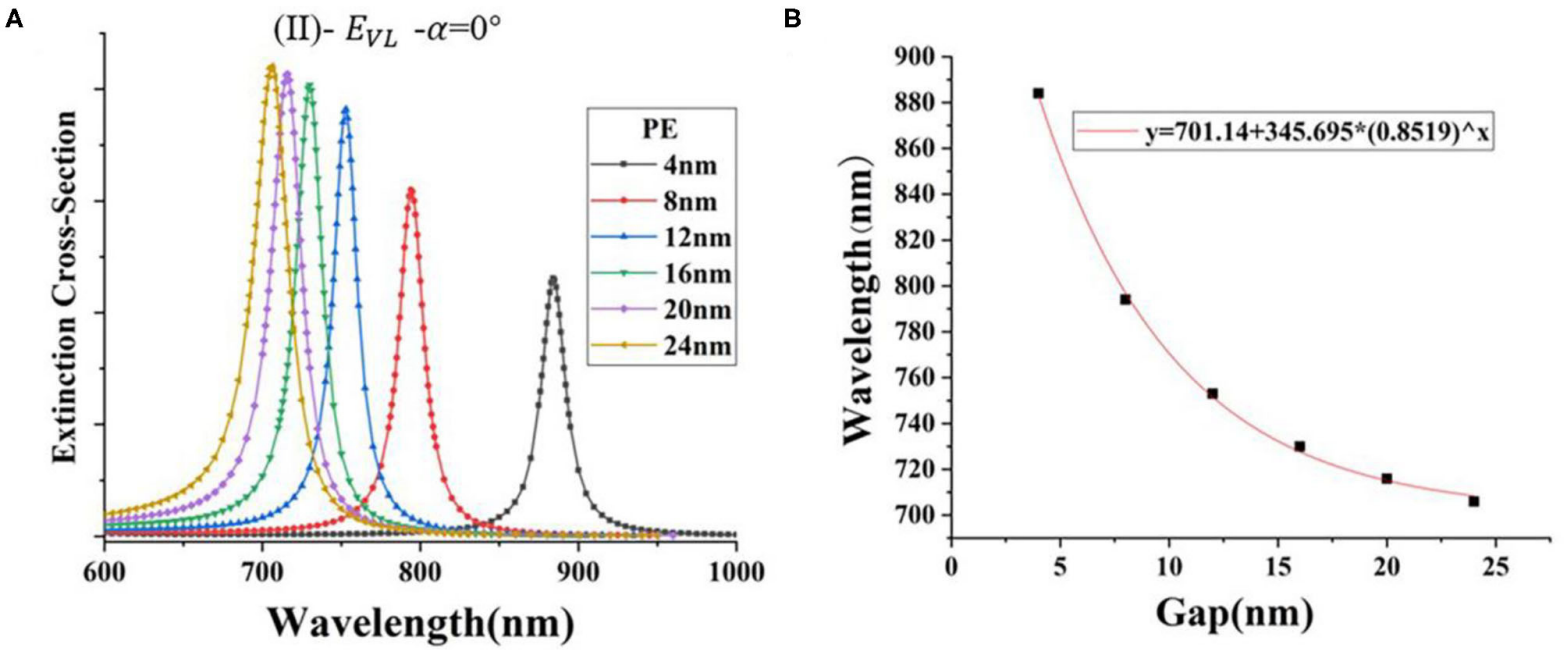
**(A)** The extinction cross-section of AgNR-film structures with different PE thicknesses; **(B)** the extinction cross-section peak position (λ) of AgNR-film structure with different PE thicknesses of the spacer layer.

**Figure 5:**
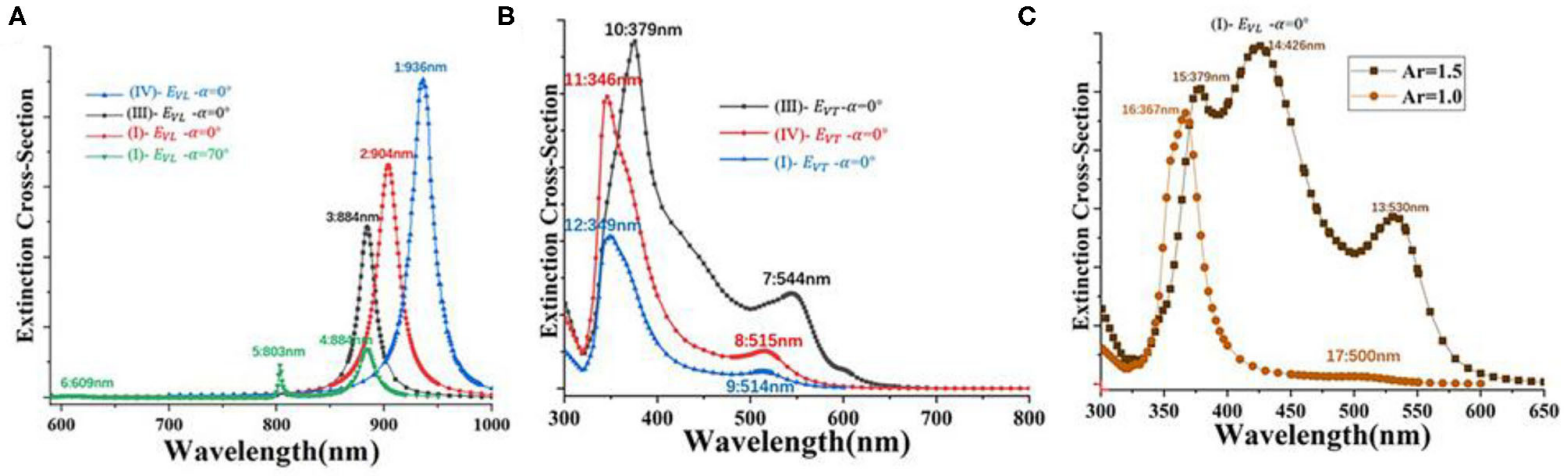
**(A)** Extinction cross-section of different nanostructures (I, III, IV) under *E*_*VL*_ and incident light with different incidence angles (α = 0° or 70°), *A*_*r*_ = 3.5. **(B)** Extinction cross-section of different nanostructures (I, III, IV) for a constant *E*_*VT*_ with incidence angles α = 0°, *A*_*r*_ = 3.5. **(C)** *A*_*r*_ = 1.0, 1.5 in Figure 2A.

Second, in the Figure 1 (II) model, the incidence angle is α = 0° and the electric field is *E*_*VL*_ (azimuth angle = 0°). The space layer PE is added to the system with the highest FOM (AgNR-film with *A*_*r*_ = 3.5 in the air), and the thickness of PE is changed evenly. The refractive index (*n*_*a*_) of PE and the thickness of the double layer measured by ellipsometry are 1.58 and 4 nm, respectively (Mock et al., 2008). Figure 4 shows that, when the thickness of the PE layer decreases, the resonance wavelength (λ) increases exponentially (Supplementary Table 4). This is because the strength of the reverse parallel mirror dipole induced on the substrate is extremely sensitive to the gap distance. The influence degree of gap materials with different refractive index (*n*_*b*_) is different; as the gap distance increases, the gap material with large refractive index will cause the resonance wavelength to move at a smaller speed (Supplementary Table 4A−8 and B-1 and Figure 5A). Fano resonance formed by combining the localized surface polaritons (LSPs) of nanorod and the surface plasmons of metal film polaritons (SPPs) is a novel method to detect small biomolecules (Eghtedari et al., 2009). The above-mentioned method is called “plasmon rulers.” This method can obtain a narrow resonance peak width; when the spectrum moves, the position of the resonance peak changes significantly, which improves the detection sensitivity. The application of this technique to the detection of DNA, RNA, and other small biological molecules has great market potential (Dathe et al., 2016).

Third, in Figure 1 (I, III, IV) model, *n*_*a*_ = 1 and the *A*_*r*_ of one/two silver nanorods is 3.5, the dimer that was arranged differently and suspending at 1.5 nm above the gold film, while the dimer between the side-by-side and the end-to-end dimer is 3 nm apart. Figure 5A shows that, when the light is vertically incident and the electric field is *E*_*VL*_, the extinction intensity and wavelength obtained by coupling two longitudinal modes (III, IV) with different arrangement modes are larger than that of a single longitudinal mode (I). There is a strong peak in the long-wave domain. When the incident light at an incident angle of 70° irradiated the monomer–gold film model and *E*_*VL*_. Thus, *E*_*VL*_ can be divided into Ex and Ez. The extinction cross-section obtained is a hybrid of the longitudinal mode and the vertical mode. There is a peak at 800 nm and a peak at 880 nm. Figure 5B shows that, when the light is vertically incident and the azimuth = 90° (*E*_*VT*_), the extinction intensity and wavelength obtained by coupling two transversal modes (III, IV) with different arrangement modes are larger than that of a single transversal mode (I), and there is a strong peak in the short-wave domain. Figure 5C shows *A*_*r*_ = 1.5 and 1, and *n*_*a*_ = 1 in Figure 2A. The extinction cross-section of *A*_*r*_ = 1.5 has three peaks, and the nanosphere has one peak in the short-wave domain. It is found that the longitudinal resonance peak shifts to the transverse resonance peak, which can be be regarded as the transition from the AgNR-film structure to AgNP film structure.

In order to find out the reason why the extinction cross-section of the AgNP film fluctuates near the wavelength of 500 nm, please refer to Supplementary Figure 6. Supplementary Figure 6C is an extended version of Figure 5C (*A*_*r*_ = 1.0) AgNP film extinction spectrum, with its absorption cross-section and scattering cross-sections added. It can be seen from Supplementary Figure 6C that the fluctuation near the wavelength of 500 nm is caused by the scattering cross-section. Supplementary Figure 6A is the spectrum cross-section of a single nanosphere of the same size and material, and it can be seen that there is no fluctuation near 500 nm. Supplementary Figure 6B reduces the original gap distance (1.5 nm) between the nanosphere and gold film to 1 nm. It can be seen that Supplementary Figure 6B has a stronger fluctuation near 500 nm than the original Supplementary Figure 6C, and this fluctuation is also caused by the scattering cross-section. Supplementary Figures 6D,E are spectum cross-sections that increase the gap distance to 3 and 5 nm. When the gap distance is 3 nm, the fluctuation at 500 nm is slightly smaller than that of the original Supplementary Figure 6C, but there is still fluctuation. When the gap distance is 5 nm, there is no fluctuation at all. Based on the above comparison, we preliminarily judge that the fluctuation of spectrum cross-section at 500 nm of Figure 5C (*A*_*r*_ = 1.0) AgNP film is due to the small gap distance between the two (<5 nm) leads to hybridization of resonance modes resulting in fluctuations in scattering.

The quantum size effect and surface effect of nanoparticles are the main factors that cause the red or blue shift of the extinction cross-section peak of nanoparticles. However, the phenomenon of the extinction cross-section peak shift of metal nanorod dimers is mainly caused by the surface effect. The main reason for the different extinction cross-section peak intensities of monomer (I) and dimers (III) and (IV) is that, compared with the case of one nanorod, the scattering effect between the two nanorods is significantly enhanced. Since the longitudinal plasma mode of nanorods has higher oscillation intensity than the transverse mode, the electric field component parallel to the length of the nanorods has the highest scattering efficiency. Therefore, the extinction cross-section of Figure 5A is stronger than that in Figure 5B. It should be indicated that when the dimers are close to each other, the dimer can be regarded as a monomer. Moreover, the electric field direction can be regarded as the long-axis direction of the nanorod. When the electric field direction is along the X-axis (*E*_*VL*_), the intensity of the end-to-end dimer is higher than that of the side-by-side dimer. It is observed that along the direction of the electric field, the side-by-side dimer can be regarded as the monomer of the nanorode with the volume increasing and the aspect ratio decreasing. However, the end-to-end dimer is the monomer of the nanorode with the volume and aspect ratio increasing. Figure 5B shows that when the electric field component is along the Y-axis (*E*_*VT*_), the strength of the side-by-side dimer (III) is higher than that of the end-to-end dimer (IV) and monomer (I) for the same reason. The transverse resonance peak is mainly controlled by the vibration of the transverse dipole, and the change in the distance between the dimers of the nanorods does not change the transverse size of the nanorods. Therefore, the short wavelength region of the domain to a four-pole model of the peak position and horizontal transverse to the dipole is located in the long-wavelength region domain model of the peak position, along with the change in the distance between the nanorods basically remaining unchanged.

**Figure 6:**
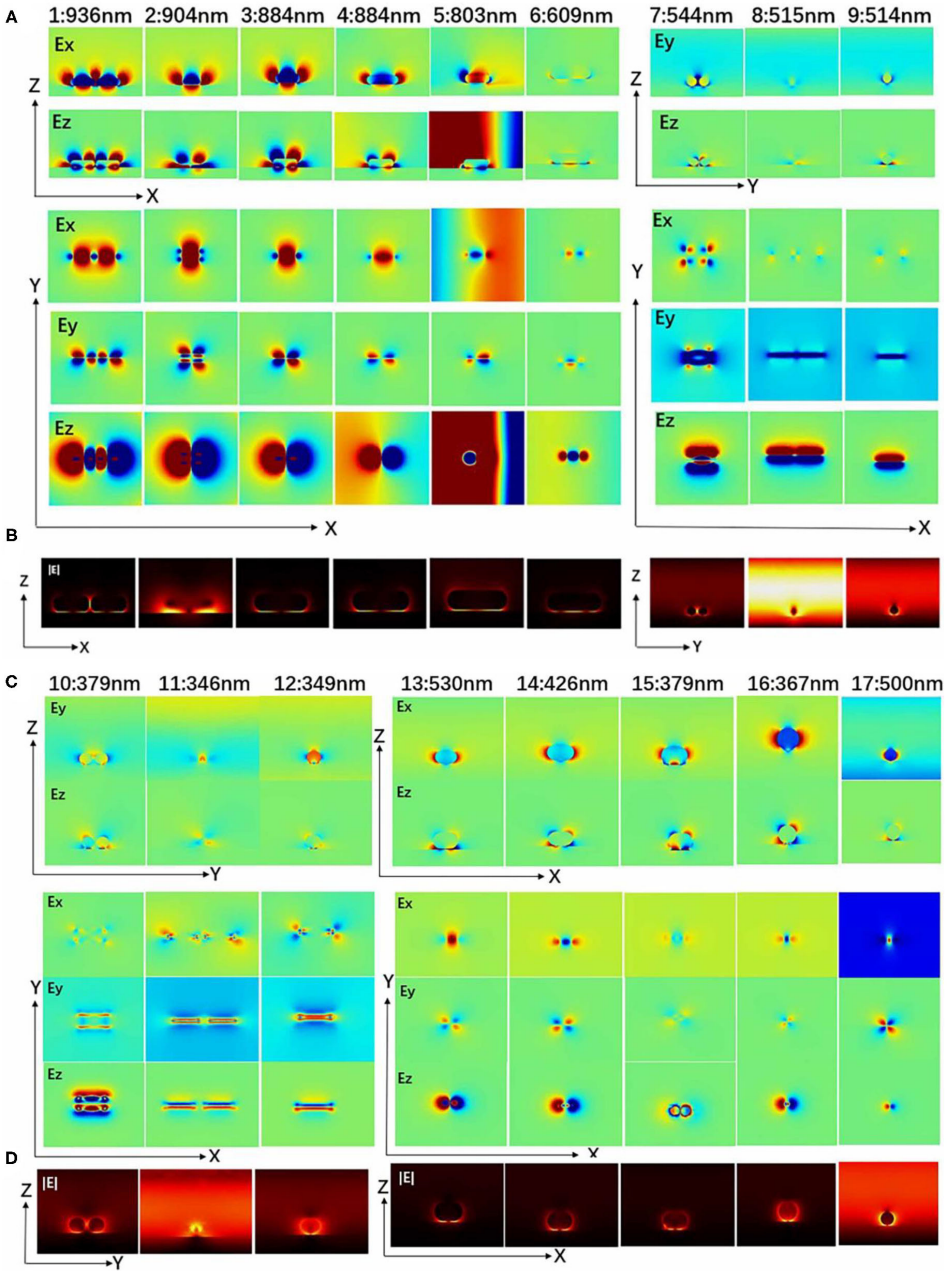
Resonance wavelength of different nanostructures (I, III, IV) of Figure 5. **(A,C)** Distribution of electric field components Ex (Ey) and Ez in the X–Z (Y, Z) plane (cut from the middle section of silver nanorods) and distribution of electric field components Ex, Ey, and Ez in the X, Y plane (cut from the interface of metal film); the above red (blue) area represents a positive (negative) electric field. **(B,D)** Electric field (**|E|**) distributions.

However, for the convenience of observation, the extinction cross-sections were normalized, respectively. The electric field diagram of (1–2) resonance wavelength in Figure 6 indicates that the (1–2) peaks in Figure 5A is the coupling of two longitudinal resonance modes with different permutations. In Figure 5A, when (I)–$E_{VL}-\alpha =70^{◦}$, the model has three extinction peaks (4–6). (4–6) are (I) nanostructures with increased frequency of extinction extremum. Moreover, Figure 6 illustrates the electric field (**|E|**) distributions and Ez in the X–Y plane of the single nanorod from two to three hotspots. It should be indicated that three hotspots with opposite vertical field components appear. They can be assigned to a waveguide mode, and their propagation in the gap is non-radiative in nature and observed as a scattering dip (Hiroshi et al., 2018). Figure 5B shows the transverse resonance mode of the model with two resonance peaks.

Figure 6 illustrates the electric field component (Ex, Ey, Ez) and electric field (|**E**|) at resonance wavelength (1–17) in Figure 5. The longitudinal surface plasma wave of the nanorods (1–3, 13–15) is excited by the electric field *E*_*VL*_. Moreover, the two ends of the silver nanorods (1–3, 13–15) show stronger local characteristics of the electric field. This indicates that a strong dipole oscillation mode along the length of the silver nanorods is excited, which produces a strong charge concentration. It is observed that the local electric field is distributed at both ends of the two silver nanorods, which leads to the enhancement of the charge resonance restoring force. In the transverse mode of (7–12), the electric field energy is concentrated on both sides of the nanorod, while the electric field at both ends approaches zero. Investigating the |**E**| of each nanorod at the resonance wavelength of (1–17), it is found that (1–5) and (13–15) peaks are two hot spots, and the peak of Figure 6A (6) has three hot spots. It is found that the new plasmon mode (6) is a higher order resonance, called a hybrid of vertical and horizontal oscillation modes. In Figure 5C, (16–17) peaks are the extinction cross-section of the nanosphere–gold film model. In Figure 6, it was observed in the (17) peak that the plasma resonance modes only respond to horizontal polarization. This distribution reveals the excited single nanospheres and dipole plasmon modes induced by the coupling between quadrupole plasmon modes. The (1–3) and (13–15) peaks are the longitudinal coupling model, (1–12) is the lateral coupling model, (4–6) is the hybridization process, and (6) is the hybrid model of vertical and longitudinal coupling. Obtained results show that silver is more convenient in terms of plasmonic applications. It is characterized by a low Ohmic loss and a large absolute value of the real part of dielectric permittivity in the visible frequency range (Song et al., 2016). This results in lower decay rates so that it is believed that silver is stronger than gold from the field localization point of view.

## Conclusion

In the present study, finite element numerical simulation is used to investigate the AgNRs film system. The simulation results show that when interacting with metal films, the anisotropic morphology makes the silver nanorods exhibit very complicated plasma hybridization characteristics. Moreover, several plasma modes with completely different resonance characteristics are excited and localized in the gap. It is observed that the redshift linearly increases as *A*_*r*_ increases. On the other hand, for the same *A*_*r*_, the position of the extinction cross-section peak changes linearly as the surrounding environment changes. Based on this silver nanorod–thin film system, plasma gas sensors can be developed for the detecting the change in specific gas concentration. When the thickness of the PE layer increases, the resonance wavelength (λ) decreases exponentially. It can be used as a plasmonic nanoruler to investigate the ultrasensitive detection of biological molecules. Therefore, it can be applied for the detection of DNA, RNA, and other small biological molecules in the future for the prevention and diagnosis of severe diseases.

## Data Availability Statement

The raw data supporting the conclusions of this article will be made available by the authors, without undue reservation.

## Author Contributions

YY and JZ designed the experiments, participated in all the experiments, analyzed the data, and wrote the draft of the manuscript. GM and ZW provided assistance to the entire experimental section and contributed in the discussion. HY contributed to the discussion of experimental results and manuscript preparation. GM and XL guided the entire experimental process and performed final revision for the manuscript. All authors contributed to the article and approved the submitted version.

## Conflict of Interest

The authors declare that the research was conducted in the absence of any commercial or financial relationships that could be construed as a potential conflict of interest.
