# Supplementary material for: Enhanced Plasmonic Resonance Characteristics of AgNRs–Gold Film Hybrid System
Source: Front Chem. 2021 Jan 21;8:553541. doi: 10.3389/fchem.2020.553541 (PMC7859493; doi:10.3389/fchem.2020.553541)
Supplement: Supplementary file 1 [file Data_Sheet_1.PDF]

Supplementary Material

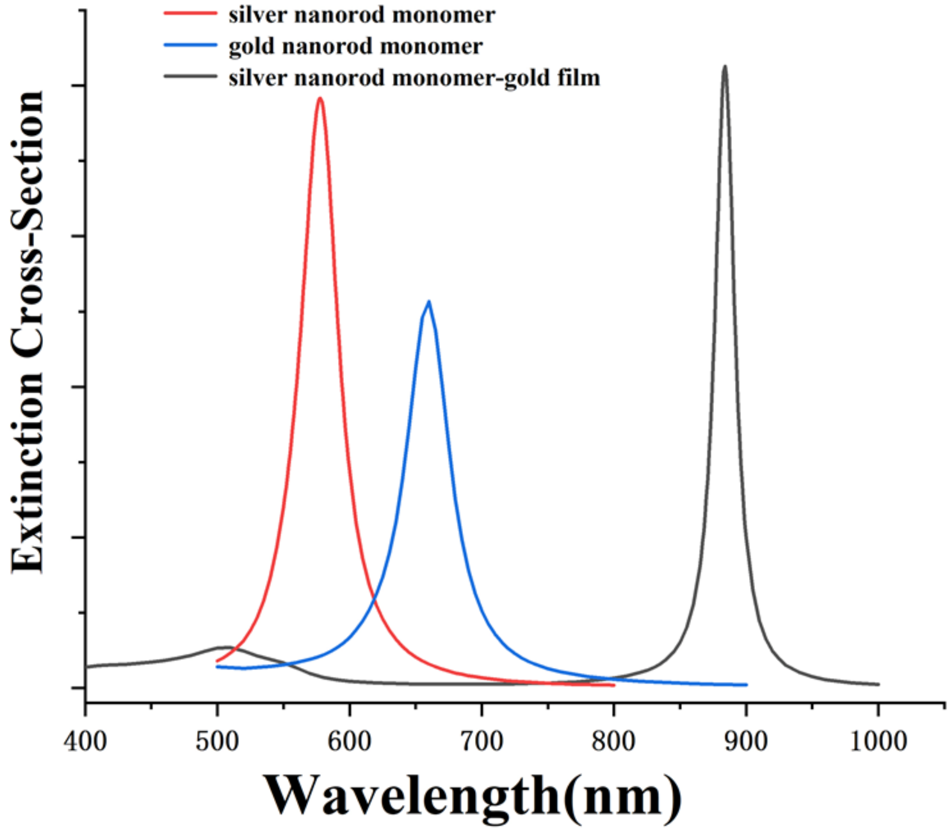

Figure 1: A_r= 3.5, normalized extinction spectra of silver nanorod monomer, gold nanorod monomer and silver nanorod monomer gold film.

Table2: In the nanostructures with different aspect ratios A_r=2.5-4.0, the deviation rate of equation 7 and 8 was calculated using the actual simulation wavelength as the reference line.

A_r	(7)/ deviation rate	(8)/ deviation rate	Simulation(λ)
2.5	675.905/-2.0%	689.315/-0.1%	690
3.0	778.01/-1.1%	789.07/0.2%	787
3.5	880.11/-0.4%	888.825/0.5%	884
4.0	982.22/-0.7%	988.58/0	989

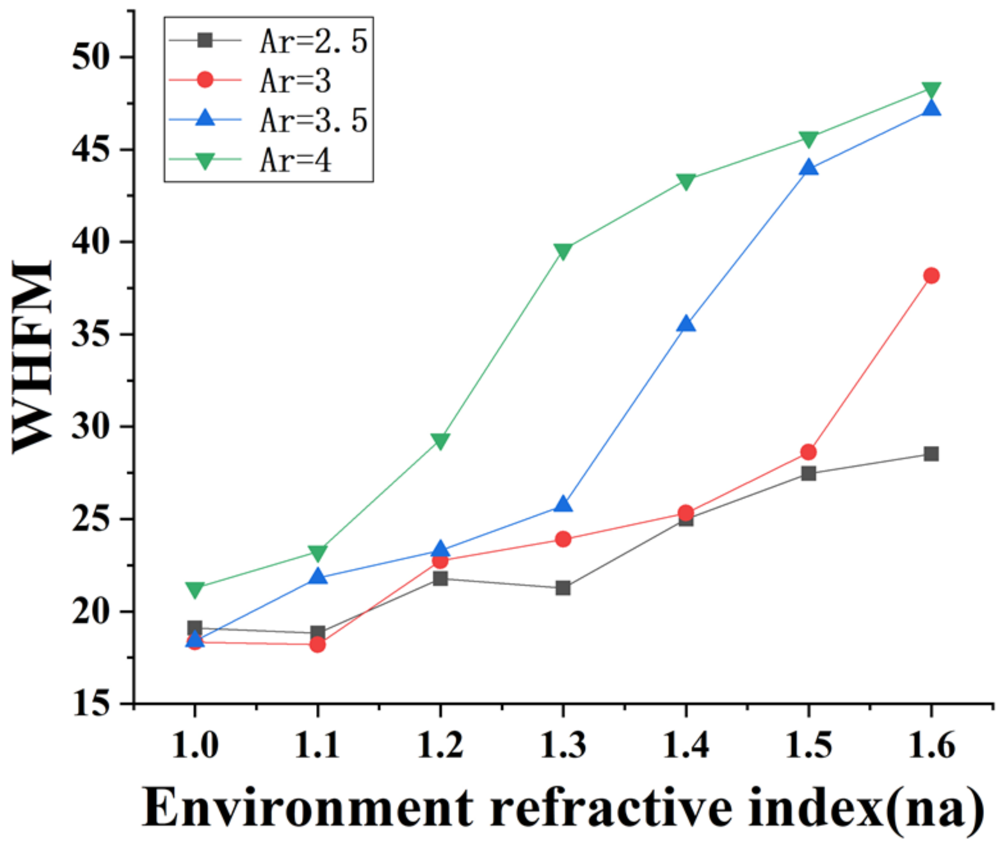

Figure 3 the full width at half maximum (FWHM) of AgNR-film structure with different A_r values in different surrounding refractive indices (n_a).

Table4: Spectral information of silver nanorod-gold film structures with different thickness of spacer layers in air

Group	A_r	Gap refractive index(n_b)	Gap distance(nm)	λ （nm）	$\Delta\lambda$ (nm)
A-1	3.5	1	1.0	959	-
A-2	3.5	1	1.5	883	76
A-3	3.5	1	2.0	841	42
A-4	3.5	1	2.5	806	35
A-5	3.5	1	3.0	783	23
A-6	3.5	1	3.5	762	21
A-7	3.5	1	3.8	753	9
A-8	3.5	1	4.0	746	7
B-1	3.5	1.58	4.0	884	-
B-2	3.5	1.58	8.0	794	90
B-3	3.5	1.58	12.0	753	41
B-4	3.5	1.58	16.0	730	23
B-5	3.5	1.58	20.0	716	14
B-6	3.5	1.58	24.0	706	10

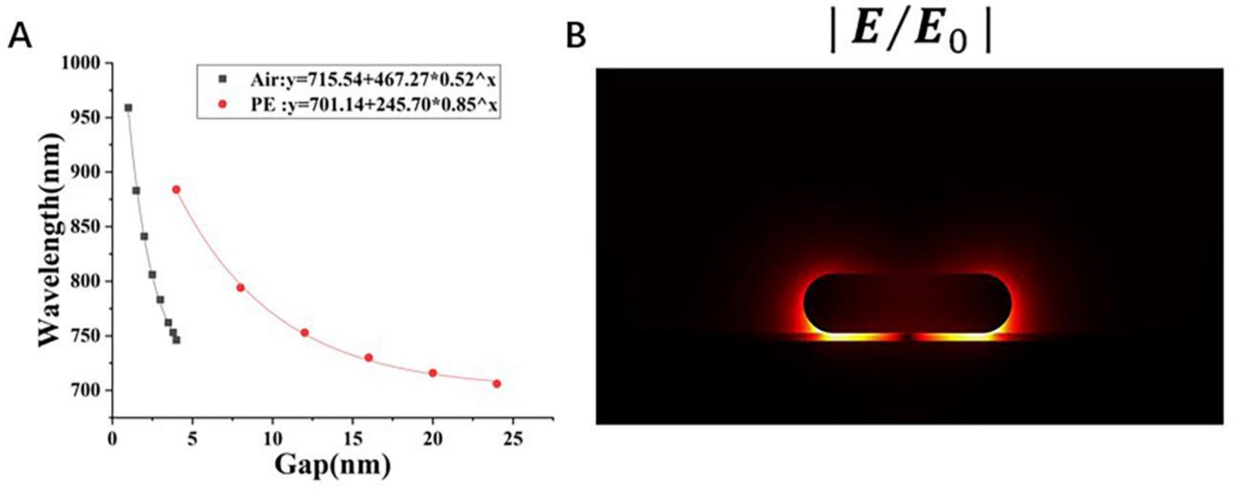

Figure 5: **(A)** The fitting functions of the relationship between the extinction cross-section peak position (λ) and Air gap distance/PE thickness; **(B)** Horizontal coupling longitudinal mode,HLM.

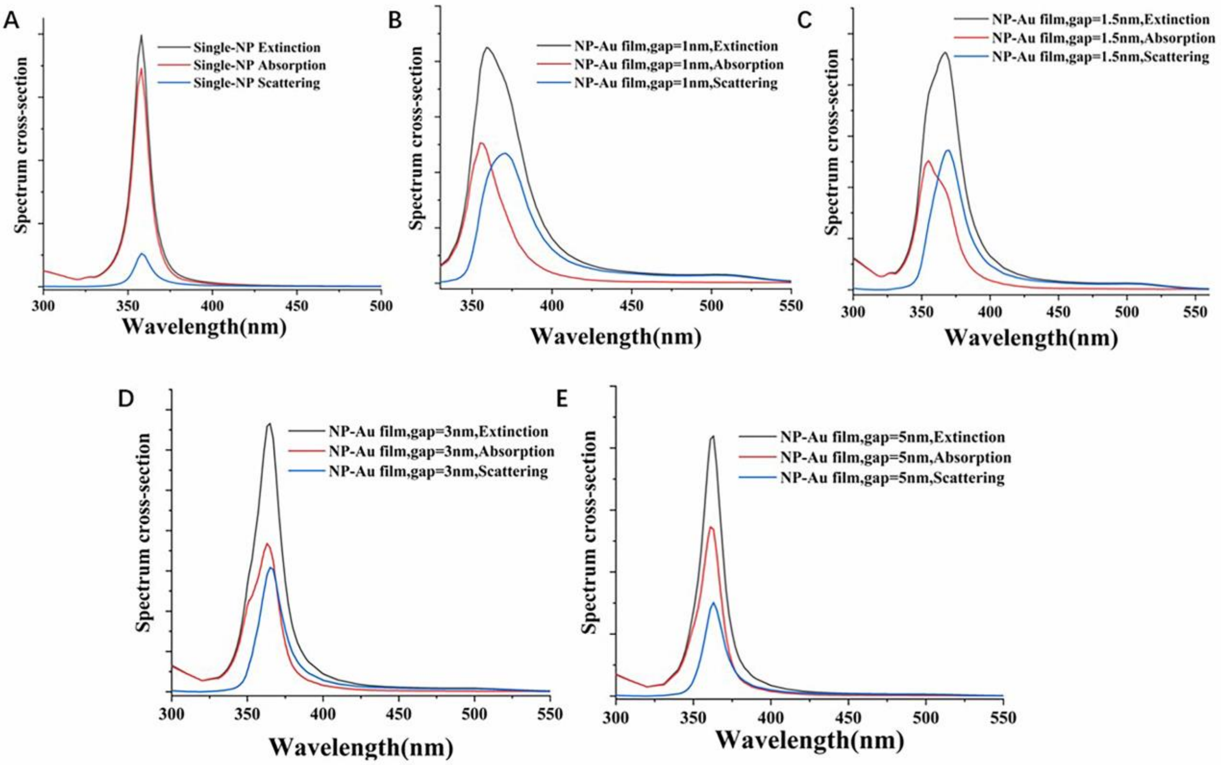

Figure 6: (A) The spectrum cross-section of a single nanosphere(Signal-NP); **(B), (C), (D)** and **(E)** are the spectrum cross-sections of the nanosphere(NP) suspended at 1nm,1.5nm,3nm and 5nm above the gold film.
